# Multidimensional spectro-temporal imaging of exciton dynamics under propagating acoustic strain in two-dimensional semiconductors

**DOI:** 10.1126/sciadv.aef5458

**Published:** 2026-08-21

**Authors:** Yuta Takahashi, Takumi Yamamoto, Hidetoshi Kanzawa, Kazuki Maezawa, Hajime Kumazaki, Yuichiro K. Kato, Shinichi Watanabe, Shun Fujii

**Affiliations:** ^1^ Department of Physics, Faculty of Science and Technology, Keio University, Yokohama, 223-8522, Japan.; ^2^ Quantum Optoelectronics Research Team, RIKEN Center for Advanced Photonics, Saitama, 351-0198, Japan.; ^3^ Nanoscale Quantum Photonics Laboratory, RIKEN Pioneering Research Institute, Saitama, 351-0198, Japan.

## Abstract

Dynamic strain offers a promising route to manipulate tightly bound excitons in two-dimensional semiconductors. Its impact, however, has so far been inferred primarily from time-averaged or spatially integrated measurements. In particular, for dynamic strain driven by surface acoustic waves (SAWs), the relatively small strain amplitude and competing piezoelectric effects have hindered direct access to the real-time evolution of exciton emission energy and recombination dynamics within a single acoustic cycle. Here we report a fully phase-synchronized, multidimensional spectro-temporal-spatial visualization of exciton emission in monolayer tungsten diselenide driven by propagating SAWs. By integrating phase-resolved microscopy and interferometric surface displacement measurements, we achieve simultaneous mapping of exciton emission energy, photoluminescence intensity, linewidth, and decay dynamics and directly correlate them with the dynamic strain field. Our work establishes propagating acoustic strain as a powerful platform for deterministic exciton control and provides a comprehensive framework for exploring nonequilibrium exciton dynamics in low-dimensional materials.

## INTRODUCTION

Two-dimensional (2D) van der Waals materials, including transition-metal dichalcogenides (TMDCs), have gained substantial attention due to their unique excitonic properties that have been revealed over the past decade. Owing to their large exciton binding energies and reduced Coulomb screening, stable excitons persist even at room temperature (*1*, *2*), enabling a variety of intriguing phenomena such as efficient light emission (*3*), strong light-matter interaction (*4*), and quantum photon generation (*5*, *6*). In particular, the realization of quantum information processing and communication has come to the forefront, as excitons in TMDCs host rich quantum degrees of freedom originating from the spin-valley locking inherent to atomically thin TMDC monolayers (*7*, *8*).

Beyond intrinsic material properties, strain engineering has emerged as a powerful and deterministic approach to manipulating excitonic states in 2D materials (*9*–*15*). Local strain potentials have been shown to induce exciton localization, giving rise to high-purity single-photon emission (*16*, *17*) as well as the manipulation of neutral excitons (*18*–*20*). Because neutral excitons are only weakly responsive to external electric fields, it is generally difficult to control exciton dynamics purely electrically. Strain, in contrast, directly modifies the band structure and exciton potential landscape, enabling efficient tuning of exciton energies as well as spatial redistribution (*21*) and transport (*22*, *23*) without introducing chemical disorder (*24*) or extrinsic defects (*25*).

Surface acoustic waves (SAWs), especially Rayleigh SAWs, are elastic waves propagating along a solid surface. The dynamic and spatially periodic strain field directly couples to excitonic states and charge carriers in 2D systems (*26*–*32*) and nanostructured semiconductors (*33*–*37*). Over the past decade, SAWs have been demonstrated to control exciton interactions, where propagating acoustic waves can modulate exciton emission energies and intensities and can induce exciton transport through strain and piezoelectric effects (*26*–*28*, *31*–*33*, *35*). While static strain engineering has provided deep insights into exciton localization, funneling, and transport in 2D semiconductors (*18*, *22*, *38*), often enabled by large strain magnitudes exceeding several percent, the exploration of ultrafast, dynamic strain driven by SAWs remains comparatively limited. In particular, the relatively small strain amplitudes generated by SAWs (typically below ∼0.1%) are frequently masked by accompanying piezoelectric fields, making it nontrivial to visualize and fully exploit pure strain-induced exciton dynamics.

A variety of optical imaging and spectroscopy approaches have been used to investigate SAW-driven exciton dynamics in TMDCs, including spatially resolved photoluminescence (PL) measurements (*28*) and phase-synchronized excitation schemes (*39*). These studies have primarily focused on exciton transport and density redistribution, revealing that exciton flux can be guided along the SAW propagation direction and that, in some cases, changes in emission energy and intensity accompany this transport (*28*, *39*). However, the weak coupling between excitons and the acoustic wave, mainly arising from a velocity mismatch, limits the efficiency of transport and obscures how exciton recombination is influenced by the underlying band structure modulation.

Despite such extensive studies on SAW-driven exciton dynamics, it remains unclear how multiple competing mechanisms, including strain-induced band modulation, piezoelectric fields, exciton funneling and dissociation, as well as many-body interactions, collectively govern exciton properties within a single acoustic cycle during the transient evolution and decay of the exciton population. Existing approaches access only a subset of exciton observables, and the simultaneous real-time evolution of exciton emission energy, intensity, and decay dynamics under a dynamically varying strain field has not yet been directly accessed. As a result, how transient bandgap modulation by SAW strain governs exciton recombination and relaxation dynamics in TMDCs remains an open question.

Here, we address this question by implementing a fully phase-synchronized and spectro-temporal-spatially resolved PL imaging platform, combined with an ultrasensitive surface displacement measurement. This approach enables clear visualization of transient bandgap modulation and exciton dynamics driven by propagating Rayleigh SAWs in monolayer tungsten diselenide (WSe_2_). We directly correlate a periodic dynamic strain profile with the exciton emission energy, intensity, and linewidth through stroboscopic observations at room temperature. Furthermore, time-resolved PL (TRPL) measurements reveal the transient exciton dynamics under periodic strain potentials, which are quantitatively explained by a theoretical model incorporating strain-dependent intervalley scattering. Last, by performing time-energy–resolved spectroscopy, we achieve a comprehensive visualization of exciton dynamics across all relevant dimensions. This multidimensional imaging approach provides a complete picture of SAW-driven exciton dynamics in 2D semiconductors, where the high-frequency dynamic potential usually hinders intuitive visualization. This work also presents important insights for exploring long-range transport and deterministic control of neutral excitons in 2D systems, leading to future deployment of energy-efficient optoelectronic devices.

## RESULTS

### Concept and universal PL properties under the SAW

**Fig. 1.**
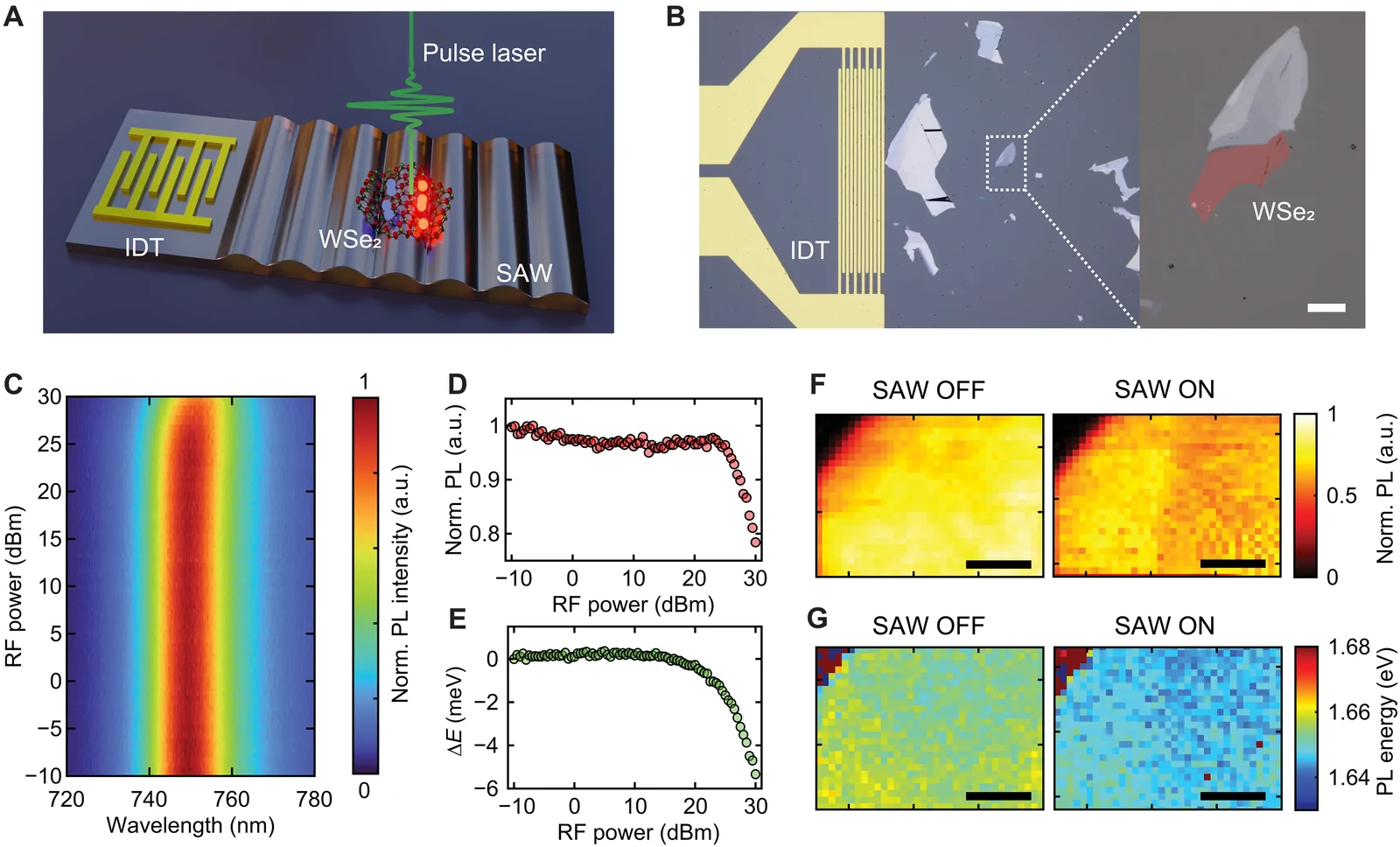
Exciton emission modulation of monolayer WSe_2_ by SAWs. (**A**) Conceptual illustration of dynamic modulation of monolayer WSe_2_ by propagating SAWs. (**B**) Optical micrograph of the WSe_2_ flake integrated on the SAW device. The monolayer region is highlighted in red. Scale bar, 10 μm. (**C** to **E**) PL modulation under increasing RF power applied to the SAW device. Both the PL intensity and emission energy decrease substantially when the RF power exceeds ∼20 dBm (100 mW). (**F** and **G**) Spatial maps of integrated PL intensity and emission energy with and without SAW excitation at an RF power of 30 dBm. The PL quenching is attributed primarily to exciton dissociation driven by the in-plane piezoelectric field associated with the SAW, whereas RF-induced heating contributes dominantly to the time-averaged spectral red shift. Scale bars, 2 μm. a.u., arbitrary units.

The conceptual illustration of the dynamic modulation of monolayer WSe_2_ by acoustic strain is shown in Fig. 1A. A mechanically exfoliated monolayer WSe_2_ is placed on a 128° Y-cut LiNbO_3_ substrate patterned with a pair of interdigital transducers (IDTs). When driven with a radio frequency (RF) microwave signal, the IDTs generate SAWs that propagate along the substrate and periodically deform the atomically thin WSe_2_ layer. This mechanical perturbation and the piezoelectric field dynamically modulate the optical response of the material through band structure modification. A micrograph of the fabricated device is shown in Fig. 1B (see Materials and Methods for details of the fabrication process).

We first investigate the general behavior of PL emission in monolayer WSe_2_ under the influence of a SAW. A 532-nm pulsed laser is used for optical excitation, and the SAW frequency $f_{\text{SAW}}$ is set to ∼377 MHz, which corresponds to approximately the fifth harmonic of the laser repetition rate $f_{\text{rep}}$ but without strict synchronization, resulting in a quasi-continuous-wave (CW) excitation condition. We measure PL spectra while gradually increasing the RF power to directly visualize the SAW-induced PL modulation (Fig. 1, C to E). As the RF power increases, the PL intensity exhibits pronounced quenching, accompanied by a clear spectral red shift. Under an RF power of 30 dBm and an optical excitation power of 1 μW, we observe PL quenching of up to 25% and a red shift of ∼6 meV. Spatial maps of the integrated PL intensity and emission energy with and without SAW excitation are presented in Fig. 1 (F and G), respectively.

The PL quenching is attributed primarily to exciton dissociation driven by the in-plane piezoelectric field associated with the SAW, which can spatially separate bound electron-hole pairs and suppress radiative recombination (*27*, *31*, *40*). In contrast, the origin of the time-averaged spectral red shift requires more careful consideration. Although piezoelectric Stark shifts have been reported in related SAW-driven TMDC systems (*27*, *40*) and III-V semiconductors (*41*), our control measurements indicate that RF-induced heating provides a dominant contribution to the red shift under the present quasi-CW conditions, consistent with previous reports where acoustic heating also affected the PL response (see Materials and Methods and the Supplementary Materials) (*42*–*44*).

From these results, we confirm that monolayer WSe_2_ is subjected to strong piezoelectric fields and RF-induced heating when the RF power is sufficiently high. Under such conditions, the electric field seems to dominate the PL quenching through exciton dissociation, while RF-induced heating contributes to the time-averaged spectral red shift. In principle, the dynamic strain should modulate the PL properties depending on the phase of the Rayleigh SAW; however, this effect is not visible in the current measurement. Time- and spatially averaged PL spectra therefore provide only limited information and obscure the real-time dynamics of bandgap modulation under acoustic strain.

### Phase-synchronized spectroscopy and surface displacement measurements

**Fig. 2.**
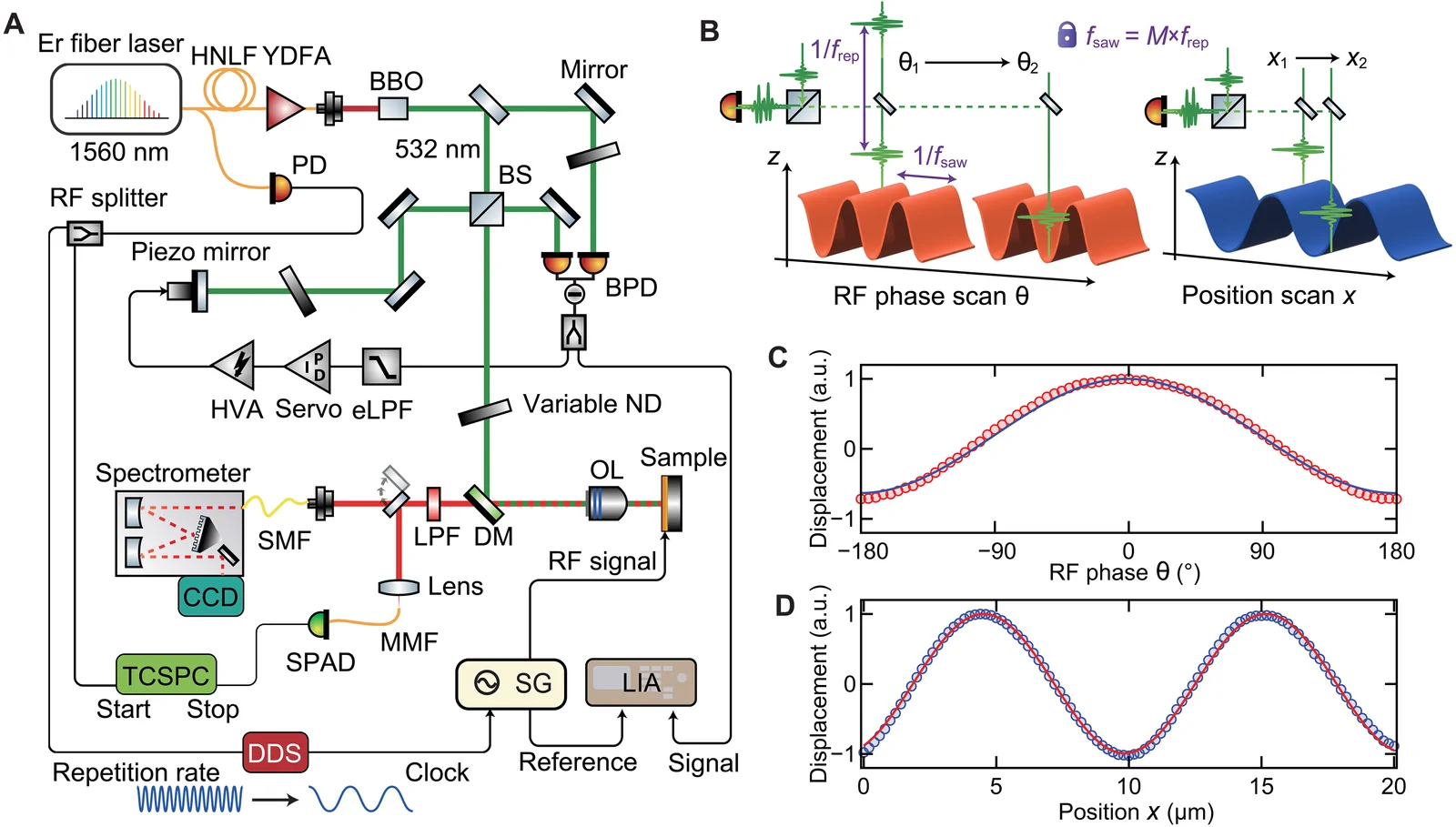
Fully phase-synchronized multidimensional measurement system. (**A**) Experimental setup. A frequency-converted 532-nm femtosecond pulse laser is used as the excitation source, and microspectroscopy and TRPL measurements are performed under phase synchronization between the pulse repetition rate and the SAW frequency. An actively stabilized Michelson interferometer is implemented for ultrasensitive surface displacement measurements. HNLF, highly nonlinear fiber; YDFA, ytterbium-doped fiber amplifier; BBO, β-barium borate crystal; (B)PD, (balanced) photodetector; BS, beam splitter; ND, neutral density filter; DM, dichroic mirror; OL, objective lens; LPF, long-pass filter; SMF, single-mode fiber; MMF, multimode fiber; CCD, charge-coupled device; SPAD, single-photon avalanche detector; TCSPC, time-correlated single-photon counting; DDS, direct-digital synthesizer; SG, signal generator; LIA, lock-in amplifier; eLPF, electrical low-pass filter; HVA, high-voltage amplifier. (**B**) Schematic of interferometric surface displacement measurements. Under the phase-synchronized condition, $f_{\text{SAW}}=M\times f_{\text{rep}}$ (where *M* is an integer), the interference signal directly reflects the out-of-plane surface displacement, which is retrieved by lock-in detection. (**C** and **D**) Measured surface displacement and corresponding fits obtained by scanning the RF phase and the sample position, respectively. The extracted spatial period of the sinusoidal profile agrees well with the SAW wavelength.

To overcome the limitations of conventional, time-averaged measurements, we develop a fully phase-synchronized PL spectroscopy setup (Fig. 2A). The key requirement of this approach is to exactly match $f_{\text{SAW}}$ to an integer multiple of the laser repetition rate $f_{\text{rep}}$ (the fifth harmonic in this work). This condition enables precise timing synchronization between optical excitation and the spatial phase of the propagating SAW, allowing us to selectively acquire PL signals corresponding to a specific acoustic phase. In addition, we generate a 10-MHz reference clock directly from the laser repetition rate using a direct-digital synthesizer. This reference disciplines the RF signal generator for SAW excitation, ensuring long-term phase stability between the SAW and the pulsed excitation, although the seed laser itself is free running.

The optical setup consists not only of a confocal spectroscopy system but also of a phase-sensitive surface displacement measurement based on a pulsed-laser Michelson interferometer (*45*, *46*). The measurement arm probes the out-of-plane motion of the SAW through a microscope objective, and the reflected light is interferometrically combined with the reference arm before photodetection. The relative optical path length is finely controlled using a piezo-mounted mirror to maximize fringe visibility, and a closed-loop feedback stabilizes the interferometer, suppressing slow drift and phase fluctuations (see Materials and Methods for more details).

We retrieve the out-of-plane displacement via lock-in detection of the interference signal, acquiring the vibration profile either by scanning the RF phase of the signal generator or by spatially scanning the sample (Fig. 2B). A detailed derivation of the SAW displacement using lock-in detection is provided in the Supplementary Materials. Figure 2 (C and D) shows the displacement waveforms and the fitting results. Notably, the measured period of the sinusoidal profile (∼10.6 μm) agrees well with the expected SAW wavelength $\lambda_{\text{SAW}}=v/f_{\text{SAW}}$, where *v* = 3979 m/s is the sound velocity of LiNbO_3_. Additional results of the position scan measurements at different RF phases are shown in fig. S3. This measurement provides a powerful tool for identifying strain profiles of propagating SAWs with high spatial and phase resolution, which is essential for quantitatively correlating dynamic strain with exciton emission dynamics.

### Visualization of spatio-spectrally resolved exciton emission properties

**Fig. 3.**
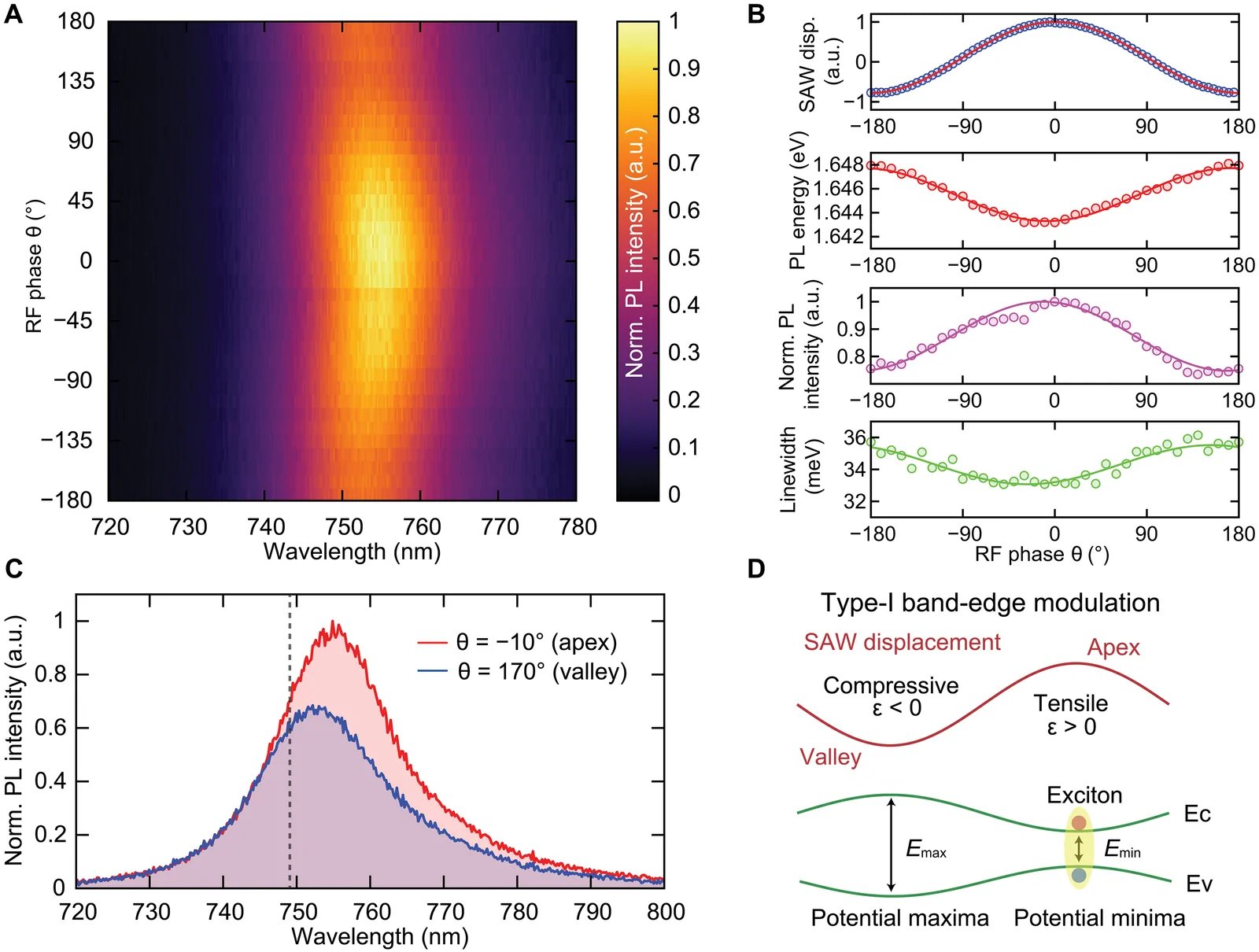
Spatio-spectral visualization of PL modulation by dynamic strain. (**A**) Measured PL spectrogram obtained by scanning the RF phase over one SAW period, where the RF input power is 30 dBm. (**B**) Evolution of the SAW displacement, PL peak energy, intensity, and linewidth as a function of the RF phase. Solid lines represent sinusoidal fits. (**C**) Representative PL spectra at RF phases of −10° and 170°, corresponding to the apex and valley of the SAW, respectively. The gray dashed line indicates the neutral PL peak position in the absence of SAW excitation. (**D**) Schematic illustration of type-I band-edge modulation induced by the SAW. The valence and conduction band edges oscillate out of phase with each other following the acoustic strain profile. Ec, conduction band edge; Ev, valence band edge.

With the phase-synchronized condition established, we simultaneously measure the PL spectrum and the surface displacement while gradually sweeping the RF phase. Figure 3A visualizes the dynamic modulation of exciton emission over a SAW cycle, where enhanced PL coincides with a spectral red shift, while suppressed emission is accompanied by a blue-shifted peak. Further spectral analysis reveals a clear correlation between PL energy, intensity, and linewidth and the measured surface displacement (Fig. 3B). We note a slight phase offset between the SAW displacement and the PL properties, which is likely attributable to spatial averaging over the finite laser spot size, comparable to ∼5% of the SAW wavelength, as well as to uncertainties in spectral peak extraction at limited signal-to-noise ratios.

To understand the dynamic behavior of exciton emission, we compare PL spectra at the apex (θ = −10°) and the valley (θ = −170°) of the SAW (Fig. 3C). At the tensile apex, the PL peak becomes brighter and spectrally narrower, whereas at the compressive valley it becomes darker and broader, corresponding to red- and blue-shifted energies, respectively. The gray dashed line in Fig. 3C marks the PL energy before SAW excitation and serves as a reference for evaluating the red- and blue-shifted states. Although the average PL energy shifts away from this neutral point due to acoustic heating at high RF power, we still observe a clear phase-dependent oscillation of both the peak energy and emission intensity.

The piezoelectric field associated with the SAW oscillates at the SAW frequency and is intrinsically phase dependent (*33*), whereas different physical responses to this field could exhibit distinct frequency signatures. For example, the Stark response is governed by the squared electric field and would give rise to an energy shift at twice the SAW frequency (*41*). However, the observed PL energy modulation (Fig. 3B) is well reproduced by a single-frequency (1 *f*_SAW_) sinusoidal function, without requiring a second-harmonic component. This suggests that the Stark contribution remains limited under the present phase-synchronized conditions. Thermal PL shifts due to RF heating evolve on a timescale much slower than the SAW period and thus appear as a phase-independent background. While electric field–induced processes, including Stark shifts and exciton dissociation, may in principle produce phase-dependent signatures, their signatures are not clearly resolved under the present experimental conditions.

Here, we primarily explain the observation in terms of type-I band-edge modulation induced by the dynamically varying strain field. The band diagram under SAW excitation is shown in Fig. 3D. In this configuration, the valence band edge oscillates in phase with the SAW displacement, whereas the conduction band edge varies out of phase. As a result, the bandgap energy follows the temporal profile of the Rayleigh SAW, leading to a periodic modulation of the excitonic emission energy synchronized with the acoustic phase. From the total amplitude of bandgap modulation (Δ*E*_g_ = 4.42 meV) and the reported strain sensitivity of monolayer WSe_2_ *A* exciton (∼−49 meV/%) (*47*), we estimate the maximum dynamic strain induced by the SAW to be ε_0_ = 0.045%. Although this value is smaller than typical static strain levels used in strain-engineering studies (*22*, *38*), it is within the range expected for SAW-driven dynamic strain fields in monolayer TMDCs (*27*, *39*, *44*, *48*). This indicates that the observed dynamic behavior is consistent with strain-induced band-edge modulation.

At high excitation densities, the PL intensity is gradually saturated by exciton-exciton annihilation (*49*), while the bandgap modulation amplitude remains unchanged, indicating that the spectral shift is governed by type-I modulation (figs. S4 and S5). We also confirm that the variation of the PL energy is proportional to the square root of the RF power, as shown in fig. S6. This behavior is consistent with the fact that the SAW amplitude scales linearly with the RF input voltage (see also fig. S7) (*50*).

In addition to the emission energy, the PL intensity and linewidth also show phase-dependent modulation synchronized with the SAW displacement. We observe enhanced PL at the apex, where tensile strain reaches its maximum, consistent with observations under static strain (*22*). This behavior is well explained by a strain-induced energy gradient that funnels excitons toward the tensile regions, increasing the local exciton density and thus the emission efficiency (*18*). It is also noteworthy that valley-generated excitons can drift toward neighboring apex positions, driven by this potential gradient, where they eventually recombine radiatively. Such exciton funneling is expected to occur particularly when the excitation spot lies on a slope between neighboring potential minima.

However, the simple funneling picture alone would suggest that excitons could also accumulate in regions where the potential gradient becomes weak, such as around the compressive valley, potentially leading to PL emission comparable to or even stronger than that observed on the SAW slopes. The fact that we observe strong intensity suppression instead represents a clear deviation from this funneling-only interpretation. The opposite trend observed here, namely, PL quenching and linewidth broadening at the valley, indicates that additional scattering channels dominate in this regime.

These behaviors can be attributed to enhanced intervalley exciton scattering under compressive strain. The phonon-assisted transition from the direct (*KK*) to indirect (*KQ*) valleys accelerates nonradiative relaxation and linewidth broadening. Conversely, tensile strain destabilizes Q-valley excitonic states through weaker exciton-phonon coupling (*10*, *47*, *51*), thereby enabling more efficient radiative recombination. We observe that the linewidth varies in the opposite phase to the PL intensity (bottom of Fig. 3B), consistent with this scattering-based interpretation. This mechanism explains both the reduced (enhanced) PL intensity and the broader (narrower) linewidth at the valley (apex) position, in agreement with strain-dependent intervalley dynamics previously reported in monolayer WSe_2_ (*22*, *47*, *51*). This *K*-*Q* intervalley scattering picture is further examined in the following TRPL measurements.

### Spatio-temporal exciton emission properties revealed by TRPL measurements

TRPL measurements provide a more complete picture of exciton emission dynamics. In the spatio-spectral spectroscopy discussed above, PL signals are integrated over the entire SAW cycle, such that any instantaneous temporal response of excitons is averaged out. It should be noted that the SAW period of 2.65 ns is comparable to the exciton lifetime in monolayer WSe_2_, implying that the exciton population evolves while experiencing a continuously varying strain field.

**Fig. 4.**
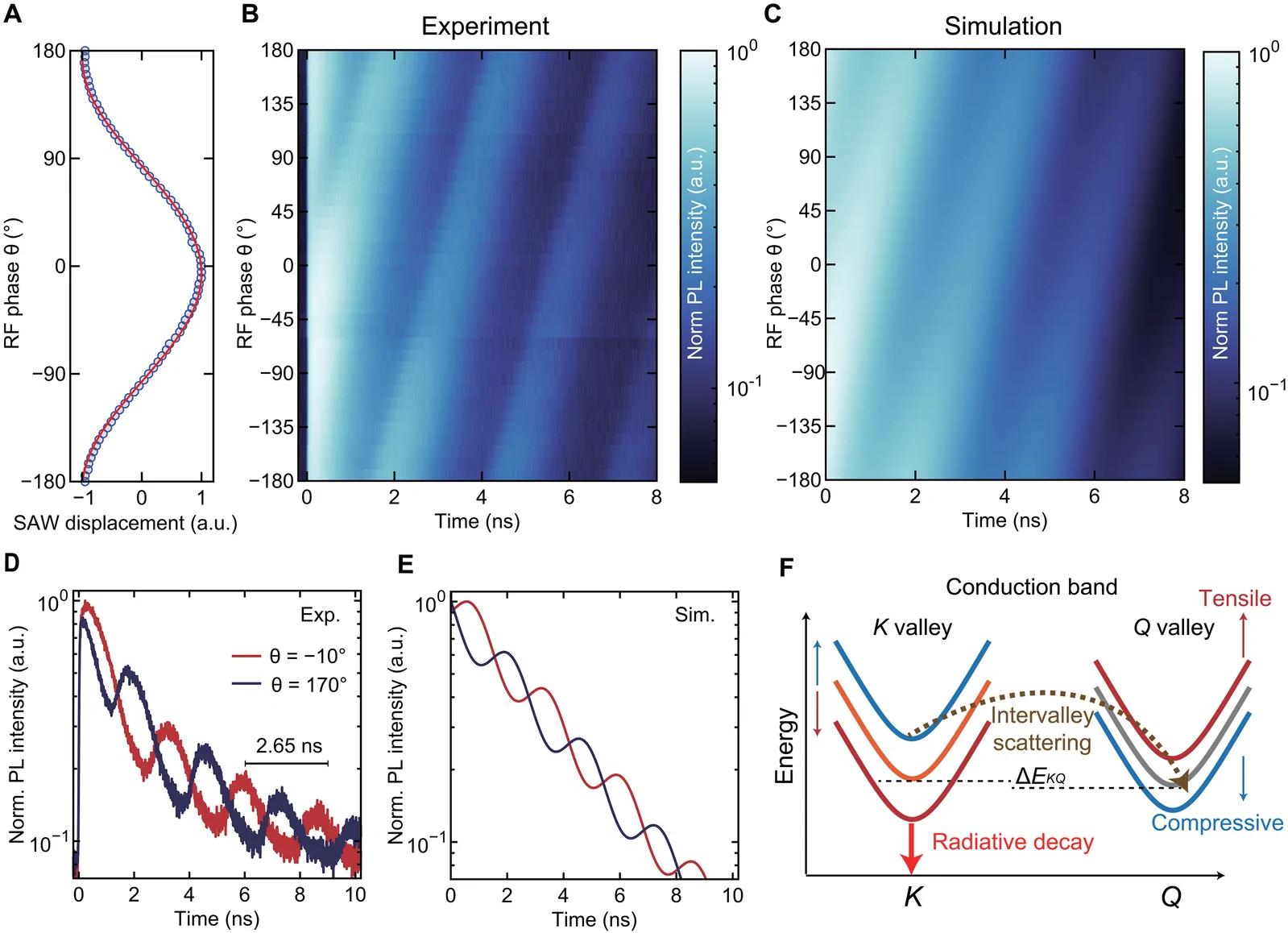
Spatio-temporal visualization of PL decay dynamics under SAW propagation. (**A**) Measured SAW surface displacement and its sinusoidal fitting obtained by scanning the RF phase. (**B**) Observed TRPL emission as a function of the RF phase, where the RF input power is 30 dBm. (**C**) Simulated TRPL emission obtained from the coupled rate equation model incorporating strain-dependent intervalley scattering. (**D** and **E**) Comparison between experimentally measured (D) and simulated (E) TRPL decay curves at representative RF phases corresponding to the tensile apex and compressive valley of the SAW. The period of the oscillation agrees with the SAW frequency (∼2.65 ns). (**F**) Schematic illustration of strain-dependent intervalley scattering between the *K* and *Q* valleys, highlighting the modulation of the energy separation Δ*E_KQ_* and its impact on radiative recombination dynamics.

We perform phase-resolved TRPL measurements while gradually sweeping the RF phase under the synchronized condition, and the surface displacement is recorded simultaneously (Fig. 4, A and B). The resulting PL decay traces exhibit a clear transient oscillation, and the oscillation period (2.65 ns) matches the SAW period. This provides direct evidence that the exciton emission is temporally modulated by the acoustic strain field. In particular, optical excitation around the tensile apex (θ = −10°) corresponds to enhanced PL emission within the SAW cycle, indicating that tensile strain increases the radiative recombination probability, as confirmed in the spectroscopy.

The strain-modulated exciton PL decay can be modeled using coupled rate equations that describe the exciton populations in the *K* and *Q* valleys (*39*)

$$\frac{{\mathrm{dn}}_{k}}{\mathrm{dt}}=-n_{k}k_{k}\left(1+\kappa_{\varepsilon }\varepsilon_{\text{saw}}\right)-n_{k}k_{\mathrm{kq}}+n_{q}k_{\mathrm{qk}}$$ (1)

$$\frac{{\mathrm{dn}}_{q}}{\mathrm{dt}}=-n_{q}k_{q}-n_{q}k_{\mathrm{qk}}+n_{k}k_{\mathrm{kq}}$$ (2)

where $n_{k\left(q\right)}$ is the exciton density in the *K*(*Q*) valley, $k_{k\left(q\right)}$ is the radiative decay rate, $\kappa_{\varepsilon }$ denotes the strain sensitivity of the decay rate, $\varepsilon_{\text{saw}}$ is the dynamic strain from the SAW, and $k_{\mathrm{kq}}$ ($k_{\mathrm{qk}}$) represents the strain-dependent intervalley scattering rate between the *K* and *Q* valleys. This model shows that dynamic strain modulates not only the radiative decay at the *K* valley but also the intervalley scattering pathway, through which bright excitons can be converted into dark excitons that do not contribute to PL emission (see Materials and Methods for details and parameters).

The simulated PL decay obtained by scanning the RF phase is presented in Fig. 4C, showing a pronounced oscillatory behavior in the PL intensity. It is noteworthy that no clear temporal oscillation arises without accounting for intervalley scattering; however, when intervalley processes are included, the simulated PL decay dynamics quantitatively reproduce the experimental observations (Fig. 4, B to E), indicating that *K*-*Q* intervalley scattering plays a critical role in the dynamic exciton emission driven by the SAW. The discrepancies observed at longer delay times could arise from slow relaxation dynamics not included in the present model, such as defect- or disorder-assisted trapping and detrapping in the nonencapsulated monolayer (*52*, *53*) and delayed population transfer involving dark or momentum-indirect exciton reservoirs (*54*, *55*). More unexpectedly, even a dynamic SAW strain as small as ∼0.05 to 0.1% is sufficient to substantially modulate the exciton emission landscape that was previously accessible only under static strain conditions (*22*).

In monolayer WSe_2_, the conduction-band minima at the *K* and *Q* valleys are nearly degenerate, and recent studies indicate that the *Q* valley lies energetically lower than the *K* valley (*51*, *56*, *57*). Because this small *K*-*Q* energy separation is highly sensitive to strain, even a weak SAW-induced strain can modulate phonon-assisted intervalley scattering between bright *K*-valley excitons and momentum-indirect *K*-*Q* excitonic states (*10*). As depicted in Fig. 4F, tensile strain lowers the conduction-band edge at the *K* valley while raising that at the *Q* valley, thereby suppressing the intervalley scattering process. This delicate balance strongly influences the exciton population dynamics and thus the PL intensity and lifetime as external strain reshapes the interband interactions.

When increasing the excitation power, the oscillatory modulation in the PL decay becomes less pronounced (fig. S8). This behavior is likely attributed to many-body interactions under high excitation, such as carrier-carrier and exciton-exciton scattering, whose rates increase with carrier density (*58*). In related systems, high carrier densities have been shown to alter intervalley scattering rates and weaken screening-dependent features in exciton dynamics (*59*). In addition, these effects tend to average out the phase-dependent recombination dynamics, consistent with the absence of screening effects in the steady-state PL spectra within the same excitation power range. We also stress that the decay dynamics are highly sensitive to dielectric conditions (*60*), as well as to residual strain (*61*) and defect density, all of which can modify intervalley scattering rates and thereby affect exciton recombination behaviors (additional TRPL simulation results assuming different initial tensile strain are also presented in fig. S9). Future studies using hexagonal boron nitride (hBN)-encapsulated samples may further clarify intrinsic strain-driven exciton dynamics. By reducing disorder, charge inhomogeneity, exciton-exciton annihilation, and piezoelectric field–induced effects, encapsulation would allow the strain-dependent modulation of *K*-*Q* intervalley scattering and other exciton responses to dynamic strain to be resolved more clearly.

### Time- and energy-resolved spectroscopy for exploring transient bandgap modulation by strain

Building on the spatially and temporally resolved measurements presented so far, we lastly move to a fully time-energy–resolved characterization to complete the picture of exciton dynamics under dynamic strain. While the phase-synchronized techniques revealed how the PL intensity and emission energy vary across the SAW cycle, they did not capture how these quantities evolve simultaneously in time and energy. This last measurement bridges that gap, allowing us to visualize the exciton response in all relevant dimensions.

**Fig. 5.**
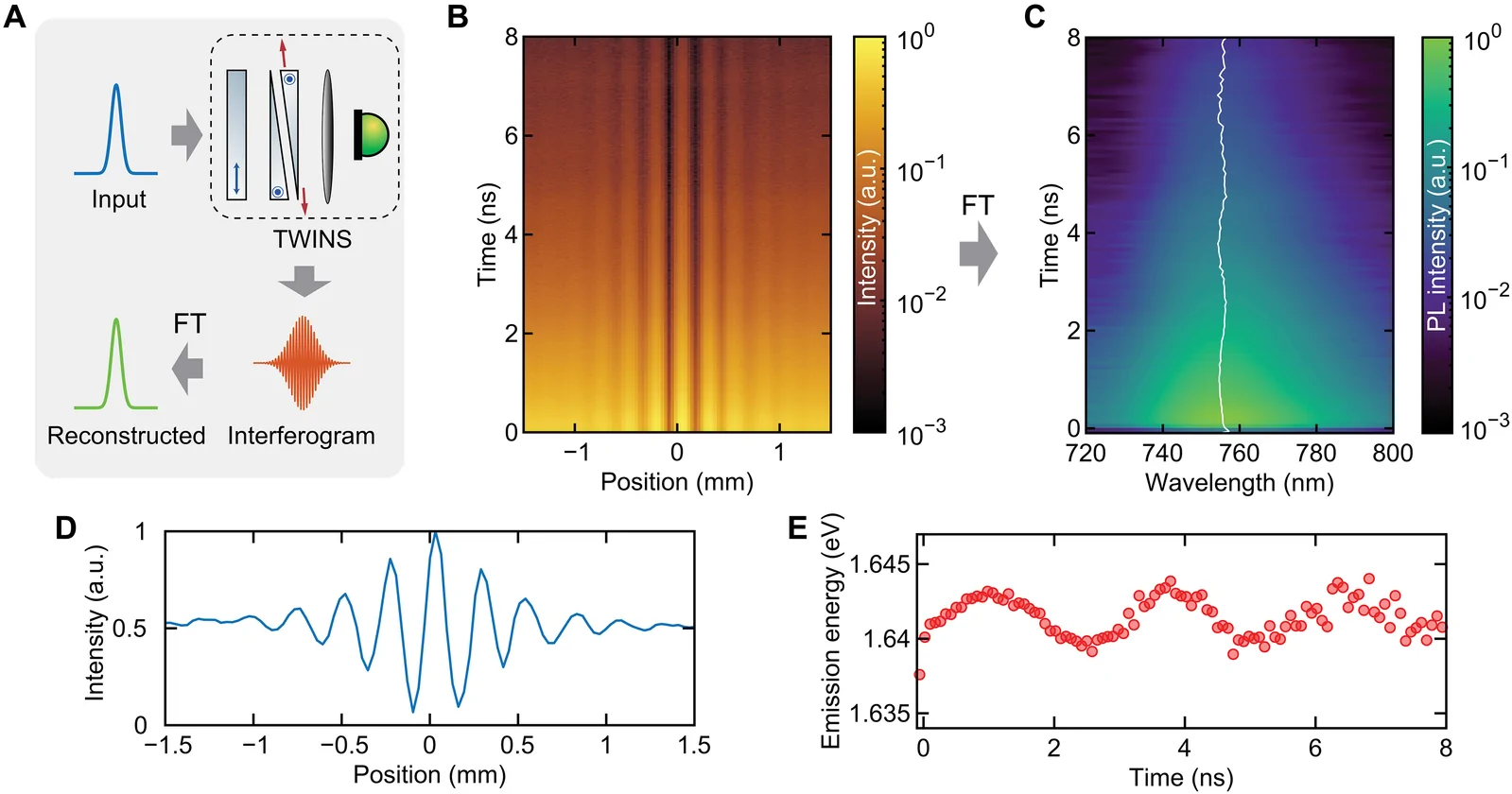
Time-energy–resolved visualization of exciton emission under dynamic strain. (**A**) Conceptual schematic of the FT interferometer. The input spectrum is reconstructed in the Fourier domain. TWINS, translating wedge–based identical pulses encoding system. (**B**) Time evolution of the interferogram signal as a function of the wedge position in the interferometer. (**C**) Reconstructed time evolution of the PL decay as a function of wavelength. The white line corresponds to the peak intensity. (**D**) Integrated interferogram signal over time obtained by scanning the wedge position. (**E**) Extracted exciton emission energy as a function of time. The oscillation period (2.65 ns) matches the SAW period, providing direct evidence of instantaneous bandgap modulation.

Here, we use a Fourier transform (FT) spectroscopy technique, in which the interference between the input light and its time-delayed replica is reconstructed as an input spectrum in the frequency domain (Fig. 5A). By combining FT spectroscopy with phase-resolved TRPL measurements, we obtain a full time-energy representation of the exciton emission under dynamic strain. While such mapping can also be achieved by spectrally filtering and recording the TRPL at each wavelength (*62*), FT spectroscopy offers a key advantage in throughput, known as the Jacquinot advantage (*63*). Moreover, this technique exploits the multiplex (Fellgett) advantage (*64*) because the full spectral information is simultaneously encoded without spatial dispersion, allowing higher photon collection efficiency for weak PL signals.

In the experiment, an FT interferometer is inserted into the optical path before PL intensity detection, and the spectral reconstruction is performed by postprocessing the recorded interferograms over time (Fig. 5, B and D). Figure 5C shows the reconstructed spectrogram of the PL decay as a function of wavelength, revealing the transient evolution of the emission energy. Unexpectedly, a transient oscillation of the PL peak energy clearly persists for up to 8 ns during the exciton decay. This behavior demonstrates that the generated excitons consistently undergo about three periods of the SAW, as expected from the picture of type-I band-edge modulation. The temporal period of this oscillation (∼2.65 ns) matches the SAW period, indicating that the exciton emission energy is modulated in real time by the propagating acoustic wave (Fig. 5E). This observation complements the phase-resolved measurements and constitutes a full spectro-temporal visualization of strain-driven exciton dynamics in 2D materials.

## DISCUSSION

In conclusion, we have demonstrated multidimensional spectro-temporal visualization of exciton emission in monolayer semiconductors under propagating strain generated by SAWs. Using a fully phase-synchronized measurement system combined with ultrasensitive surface displacement interferometry, we directly correlate the acoustic displacement with exciton emission energy, intensity, and linewidth. These measurements provide a direct visualization of the exciton response to propagating SAW fields across distinct timescales, beyond time-averaged or spatially integrated observables.

TRPL measurements further unveiled the transient exciton dynamics under periodically modulated strain potentials. The observed oscillatory behavior in PL intensity and decay dynamics is quantitatively explained by a rate equation model incorporating strain-dependent intervalley scattering, highlighting the critical role of valley dynamics in strain-driven exciton recombination. Moreover, time-energy–resolved spectroscopy based on FT interferometry enabled direct observation of transient exciton energy oscillations persisting over multiple SAW cycles.

This work provides a unified physical picture of exciton dynamics under SAW excitation, in which the observed response arises from the interplay of multiple mechanisms. Strain-induced band modulation governs the phase-resolved energy shifts, while intervalley scattering governs the phase-dependent modulation of emission intensity and linewidth, with additional contributions from strain-induced exciton funneling. Time- and energy-resolved measurements further reveal exciton dynamics across distinct timescales, showing that the emission decays while continuously responding to the dynamic strain. At the same time, we identify phase-independent contributions that do not follow the dynamic SAW modulation, such as thermally induced spectral shifts, and distinguish them from strain-driven dynamics by their slower temporal response.

This approach may further enable access to unresolved phase-dependent electric field effects arising from the complex piezoelectric fields generated by the SAW. In particular, disentangling field-induced exciton dissociation from Stark-type energy shifts, including possible distinct in-plane and out-of-plane responses, would provide deeper insight into how piezoelectric fields modulate exciton recombination and dissociation. Together, our multidimensional imaging framework establishes dynamic acoustic strain as a platform for controlling exciton dynamics in 2D semiconductors, extending static band tuning into the time domain and opening opportunities for exploring nonequilibrium exciton phenomena and engineering exciton transport and emission using acoustic waves.

## MATERIALS AND METHODS

### Fabrication of SAW devices

The SAW devices were fabricated on 128° Y-cut LiNbO_3_ substrates (Yamaju Ceramics) using a photolithography process. Hexamethyldisilane (HMDS) treatment (OAP, Tokyo Ohka Kogyo) was used to improve the adhesion between the substrate and the photoresist after prebaking at 90°C for 2 min. A photoresist (PFI-89B4, Sumitomo Chemical) was spin-coated at 3000 rpm for 30 s, followed by postbaking at 110°C for 1 min. The sample was exposed using laser lithography (DWL66+, Heidelberg Instruments) and developed using tetramethylammonium hydroxide (Tama Chemicals) for 1 min. As IDT electrodes, 3-nm Ti and 70-nm Au were evaporated using an electron beam evaporation method. The lift-off process was performed by immersing the samples in a polar solvent, *N*-methylpyrrolidone, for about 12 hours. The IDTs were electrically connected using wire bonding.

### *S*_21_ measurement of SAW devices

*S*_21_ transmission measurements using a vector network analyzer (ZNLE6, Rohde&Schwarz) were carried out to identify the resonant frequency of the fabricated devices. The two IDTs on both sides were used for the generation and detection of SAWs. Each IDT had six finger pairs, and the spacing between the fingers was 2.55 μm; each finger had a width of 2.55 μm. The fundamental resonance frequency was 365 MHz, which closely matches five times the laser pulse repetition rate (5 × 75.4 = 377 MHz). The full width at half maximum linewidth was ∼54 MHz, and this relatively broad linewidth enables resonant excitation despite the frequency detuning from the perfect resonance condition. The *S*_21_ spectrum is provided in fig. S10.

### Exfoliation and transfer of monolayer WSe_2_

WSe_2_ flakes were mechanically exfoliated from bulk crystals (HQ Graphene) using cellophane tape and transferred onto a glass-supported polydimethylsiloxane (PDMS) sheet (PF-40x40-0170-X4, Gel-Pak). The layer number was identified by optical contrast under an optical microscope. Monolayer WSe_2_ was transferred from a PDMS stamp to the target position on a SAW device using a dry-transfer technique with a home-built setup equipped with a motorized linear stage and a rotational stage.

### Discrimination between RF-induced heating and nonthermal SAW-induced effects

To distinguish RF heating–induced PL shifts from SAW-related contributions, a modulation-based control measurement was performed following previous studies (*42*, *44*). Because thermal effects evolve on a timescale much slower than the SAW period, they can be separated by modulating the optical excitation and RF drive at a much lower frequency. In this measurement, the optical excitation and RF drive were chopped at the same frequency of 100 Hz, and PL spectra were recorded for two chopping-phase conditions: The optical and RF chopping signals were either in phase or out of phase. The out-of-phase chopping condition was used as a reference for the RF heating–induced shift, whereas the in-phase chopping condition contains both SAW-related and thermal contributions. The experimental details and results are provided in the Supplementary Materials.

### Phase-synchronized PL spectrum measurements

A confocal microscopy system was used to perform phase-synchronized PL measurements at room temperature. A femtosecond pulsed laser source with a repetition rate of 75.4 MHz and a center wavelength of ∼532 nm was used to excite the *B*-exciton resonance of monolayer WSe_2_. A home-built erbium-doped fiber laser with a center wavelength of 1560 nm was used as a seed laser, and its spectrum was first broadened using a highly nonlinear fiber. The spectral component at 1064 nm was then selectively amplified by an ytterbium-doped fiber amplifier, followed by pulse compression using a transmission grating pair. Second-harmonic generation using a β-barium borate crystal enabled coherent frequency conversion from 1064 to 532 nm (*65*).

The laser beam was focused onto the sample using an objective lens with a numerical aperture of 0.8 and a working distance of 3.5 mm (LMPlanFL N 100X, Olympus). The estimated spot diameter was ∼0.5 μm, and the PL signal was collected through the same objective lens and a fiber collimator (F260FC-780, Thorlabs) after rejecting the reflected pump signal using a dichroic mirror. The collection spot size defined by the confocal plane (fiber core diameter) was ∼0.6 μm. The excitation power was 1 μW. The PL spectrum was recorded using an electron-multiplying charge-coupled device camera (iXon Ultra 888, Andor Technology) attached to a Czerny-Turner spectrometer (Kymera 193i, Andor Technology) equipped with a 300 lines/mm grating. The SAWs were driven by a signal generator (E4421B, Agilent) followed by an RF amplifier (PE15A4017, Pasternak).

### Surface displacement measurements by pulsed laser interferometry

Stroboscopic sampling with pulsed laser interferometry was used to measure the out-of-plane surface displacement of the SAW. The detected interference signal from a balanced photodetector (PDB210A/M, Thorlabs) was split into two electrical paths, one of which was sent to a lock-in amplifier (SR830, Stanford Research Systems), while the other was used for closed-loop feedback. Low-frequency amplitude modulation was applied to the RF signal for lock-in detection. The interferometer was stabilized at the quadrature point, enhancing detection sensitivity and suppressing environmental path fluctuations. A portion of the pulsed laser light was sent to the other arm of the balanced detector with appropriate power attenuation to balance the detector. While lock-in detection of the SAW displacement intrinsically suffers from a π-phase ambiguity between the peak and trough positions, this ambiguity can be resolved by exploiting the known PL red shift of monolayer WSe_2_ under tensile strain.

### Phase-synchronized time- and energy-resolved measurements

The same customized laser was used to perform TRPL measurements. The PL signal was collected using a multimode fiber with a core diameter of 50 μm and detected by a fiber-coupled single-photon avalanche detector (SPAD) module (PDM series, MPD). Time-correlated single-photon counting was performed using a time tagger (quTAG, qutools), where the photodetected laser repetition rate served as the trigger signal. The excitation power was 0.1 μW. For time- and energy-resolved measurements, an ultrastable common-path birefringent interferometer (GEMINI, NIREOS) (*66*) was inserted in front of the collection fiber. The interferogram signal was detected by the SPAD module, and the spectrum was reconstructed by Fourier transformation.

### Modeling and simulations of PL decay dynamics

The rate equations describing the PL decay dynamics (Eq. 2) were numerically solved using the Runge-Kutta method. The dynamic strain was defined as $\varepsilon_{\text{saw}}=\varepsilon_{0}\text{cos}\left(2\pi t/T_{\text{saw}}\right)$, where $\varepsilon_{0}$ is the maximum strain. The intervalley scattering rate was defined as $k_{\mathrm{kq}}=k_{\mathrm{qk}}\text{exp}\left[-\left(\Delta E_{\mathrm{KQ}}+\varepsilon_{\text{saw}}\Delta E_{\epsilon }\right)/k_{B}T\right]$, where $k_{\mathrm{qk}}=2840\;{\text{ns}}^{-1}$, $\kappa_{\varepsilon }=10\%/\%$ (*67*), $\Delta E_{\mathrm{KQ}}=-60\;\text{meV}$ (*56*), and the strain sensitivity between the *K* and *Q* valleys is $\Delta E_{\epsilon }=68\;\text{meV}/\%$ (*10*). In the simulations, $k_{B}T=25.3\;\text{meV}$, $k_{k}=2.0\;{\text{ns}}^{-1}$, $\varepsilon_{0}=0.1\%$, $T_{\text{saw}}=2.65\;\text{ns}$, and an initial tensile strain of 0.25% were assumed to fit the experimental results.
