# Supplementary material for: Multidimensional spectro-temporal imaging of exciton dynamics under propagating acoustic strain in two-dimensional semiconductors
Source: Sci Adv. 2026 Aug 21;12(34):eaef5458. doi: 10.1126/sciadv.aef5458 (PMC13496191; doi:10.1126/sciadv.aef5458)
Supplement: Supplementary file 1 [file sciadv.aef5458_sm.pdf]

Science Advances
AAAS

Supplementary Materials for

Multidimensional spectro-temporal imaging of exciton dynamics under propagating acoustic strain in two-dimensional semiconductors

Yuta Takahashi *et al.*

Corresponding author: Shun Fujii, shun.fujii@phys.keio.ac.jp

Sci. Adv. **12**, eaef5458 (2026)
DOI: 10.1126/sciadv.aef5458

This PDF file includes:

Supplementary Text
Figs. S1 to S10

Supplementary Text

Control measurement for distinguishing RF-induced heating from SAW-related effects

To evaluate the contribution of RF-induced heating to the time-averaged PL red shift observed under quasi-CW conditions, we performed a modulation-based control measurement (*42, 44*) using a different device with the same IDT design and measurement configuration as the device used in the main text. Because RF-induced thermal effects evolve on a timescale much slower than the SAW period, they can be separated from the instantaneous SAW-related response by comparing PL spectra acquired under different chopping-phase conditions. Briefly, the optical excitation and RF drive were chopped at the same frequency of 100 Hz, and PL spectra were recorded under different chopping-phase conditions. The laser light was mechanically chopped by an optical chopper, and the RF drive was directly modulated by the signal generator.

Figure S1 shows the measurement concept. In the out-of-phase chopping condition, the optical excitation probes the sample after the RF-driven excitation and is used as a reference for the slow RF-heating-induced background. In the in-phase chopping condition, the optical excitation temporally overlaps with the RF drive and contains both SAW-related and thermal contributions.

Figure S2(A) shows representative PL spectra measured without SAW excitation and under the two chopping conditions. The PL peak exhibits a clear red shift in both in-phase and out-of-phase chopping conditions. As shown in Fig. S2(B) and (C), the PL intensity decreases more strongly under the in-phase condition, whereas the PL peak energy shows almost the same RF-power dependence in both chopping conditions. The difference in PL peak energy between the two conditions remains very small over the measured RF-power range (Fig. S2(D)). It should be noted that the PL intensity remains nearly unchanged in the out-of-phase chopping condition, indicating that the reduction in PL intensity caused by RF-induced heating is limited under the present conditions.

These results indicate that RF-induced heating provides a dominant contribution to the time-averaged spectral red shift under the present quasi-CW conditions. This also accounts for the large spectral offset from the PL peak position measured without SAW excitation, as shown in Fig. 3C of the main manuscript. In contrast, the additional PL quenching observed under the in-phase chopping condition is attributed to SAW-related effects, primarily exciton dissociation driven by the piezoelectric field.

Mathematical derivation of out-of-plane surface displacement via lock-in detection

The voltage of the interference signal measured by a Michelson interferometer is proportional to the surface displacement induced by the SAW and can be written as

$$I_{\mathrm{in}} = V_{\mathrm{in}} \sin(k_{\mathrm{SAW}} x - \omega_{\mathrm{SAW}} t + \theta_{\mathrm{SAW}}), \tag{S1}$$

where V_{in} is the amplitude of the interference signal, and k_{SAW}, ω_{SAW}, and θ_{SAW} are the wavenumber, angular frequency, and phase of the SAW, respectively. When amplitude modulation is applied to the SAW with an angular frequency ω_{in} and a modulation depth A, the interference signal is modulated as

$$I_{\mathrm{in,mod}} = V_{\mathrm{in}} \{(1 - A) + A \sin(\omega_{\mathrm{in}} t + \theta_{\mathrm{in}})\} \sin(k_{\mathrm{SAW}} x - \omega_{\mathrm{SAW}} t + \theta_{\mathrm{SAW}}), \tag{S2}$$

where θ_{in} is the phase of the amplitude modulation signal.

The modulated interference signal $I_{\mathrm{in,mod}}$ is then input into a dual-phase lock-in amplifier, where it is mixed with a reference signal I_{ref}. The X and Y components of the reference signal, $I_{\mathrm{ref,X}}$ and $I_{\mathrm{ref,Y}}$, are given by

$$I_{\mathrm{ref,X}} = V_{\mathrm{ref}} \sin(\omega_{\mathrm{ref}} t + \theta_{\mathrm{ref}}), \tag{S3}$$

$$I_{\mathrm{ref,Y}} = V_{\mathrm{ref}} \cos(\omega_{\mathrm{ref}} t + \theta_{\mathrm{ref}}), \tag{S4}$$

where V_{ref} is the reference amplitude, ω_{ref} is the angular frequency of the reference signal (set equal to ω_{in}), and θ_{ref} is the reference phase.

In the lock-in amplifier, high-frequency components are removed by a low-pass filter. As a result, the X and Y outputs of the lock-in amplifier are given by

$$X = I_{\mathrm{in,mod}} \times I_{\mathrm{ref,X}} \Rightarrow \frac{1}{2} V_{\mathrm{in}} V_{\mathrm{ref}} A \cos(\theta_{\mathrm{in}} - \theta_{\mathrm{ref}}) \sin(k_{\mathrm{SAW}} x - \omega_{\mathrm{SAW}} t + \theta_{\mathrm{SAW}}), \tag{S5}$$

$$Y = I_{\mathrm{in,mod}} \times I_{\mathrm{ref,Y}} \Rightarrow \frac{1}{2} V_{\mathrm{in}} V_{\mathrm{ref}} A \sin(\theta_{\mathrm{in}} - \theta_{\mathrm{ref}}) \sin(k_{\mathrm{SAW}} x - \omega_{\mathrm{SAW}} t + \theta_{\mathrm{SAW}}). \tag{S6}$$

Here, V_{in}, V_{ref}, θ_{in}, and θ_{ref} are all determined by the experimental conditions and can be treated as constants. Therefore, by sweeping the SAW phase θ_{SAW}, a sinusoidal waveform corresponding to the SAW vibration over one acoustic period is obtained by monitoring either the X or Y output of the lock-in amplifier.

Spatial profiles of traveling SAWs measured by position scan measurements

Figure S3 shows the spatial profile of the traveling SAW measured using a Michelson interferometer.

Excitation power dependence of exciton PL energy and integrated PL intensity

We performed phase-synchronized PL measurements over one SAW period at an RF power of 30 dBm under four different optical excitation powers (0.1 µW, 0.5 µW, 1 µW, and 5 µW). Figures S4 and S5 show the excitation power dependence of the modulated exciton PL energy and the integrated PL intensity, respectively.

As shown in Fig. S4, the magnitude of the exciton PL energy modulation ΔE remains nearly constant over the entire excitation power range. This result indicates that the observed energy modulation within one SAW period originates from type-I band-edge modulation, where the bandgap energy is directly modulated by the SAW-induced strain and is therefore independent of the optical excitation power.

Figure S5 presents the dependence of the integrated PL intensity on the optical excitation power. The integrated PL intensity clearly saturates at high excitation powers, suggesting a reduction in the radiative PL yield due to exciton–exciton annihilation (*49*).

Dependence of the magnitude of PL energy modulation and SAW amplitude on RF input voltage

The magnitude of exciton PL energy modulation induced by SAW strain is expected to scale with the SAW displacement amplitude, because the bandgap of monolayer WSe_2 responds linearly to applied strain, and the strain amplitude is proportional to the SAW amplitude. Therefore, by measuring the PL energy modulation while varying the RF input power, we can directly verify the linear strain-induced bandgap modulation.

Figure S6 shows the dependence of the PL energy modulation amplitude on the square root of the RF input power, which is proportional to the RF input voltage. The magnitude of the PL energy modulation increases linearly with the RF input voltage, confirming that the exciton PL energy of monolayer WSe_2 responds linearly to the SAW-induced strain.

To further validate that the measured SAW amplitude accurately reflects the surface vibration

generated by the interdigital transducer (IDT), we independently examined the dependence of the SAW amplitude on the RF input power using the optical interferometer. Since the SAW amplitude generated by the IDT is expected to be proportional to the RF input voltage (i.e., to the square root of the RF input power), we measured the SAW amplitude while sweeping the RF input power.

Figure S7 shows the dependence of the SAW amplitude on the square root of the RF power. The SAW amplitude increases nearly linearly with the RF input voltage, demonstrating that the interferometrically measured SAW amplitude reliably reflects the magnitude of the surface vibration generated by the RF-driven IDT.

RF phase dependence of exciton PL time evolution at five different optical excitation powers

We conducted phase-synchronized transient PL intensity measurements over one SAW period, as shown in Fig. 4 of the main text, with an RF power of 30 dBm at five different optical excitation powers (0.05 µW, 0.1 µW, 0.5 µW, 1 µW, and 5 µW). Figure S8 shows the results of the time-resolved PL measurements. As the excitation power increases, the oscillatory modulation in the PL decay becomes less pronounced.

Simulation of PL decay dynamics with different initial strain levels

Figure S9 shows the simulated time-resolved PL under the dynamic strain field (strain amplitude of 0.05%) with different initial strain levels ranging from 0% strain to 1% tensile strain, based on Eqs. (1) and (2) in the main text. All other parameters are the same as those used in the main text. The results clearly show that the magnitude of the periodic oscillation of the transient PL intensity induced by the SAW decreases as the initial strain level increases. This indicates that intrinsic strain affects the intervalley scattering rates between the K and Q valleys (*22, 47, 51*), resulting in different exciton recombination dynamics even under the same SAW strain field.

S_{21} response of the SAW device

Figure S10 shows the transmission spectrum ($|S_{21}|$) of the SAW device used in this work.

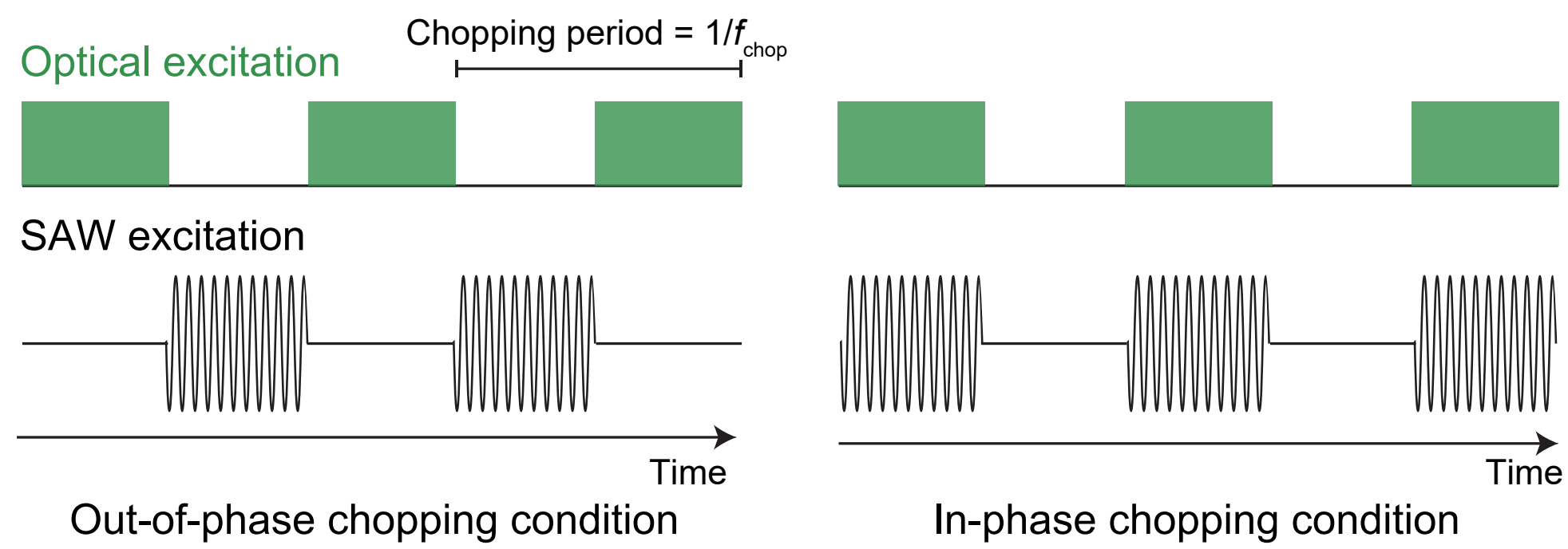

Figure S1: **Schematic of the modulation-based control measurement used to discriminate RF-induced heating from SAW-related effects.** The optical excitation and RF drive were chopped at the same frequency, f_{chop} = 100 Hz. In the out-of-phase chopping condition, the optical excitation probes the sample after the RF-driven SAW excitation, providing a reference for the slowly varying thermal background. In the in-phase chopping condition, the optical excitation temporally overlaps with the RF drive and therefore contains both SAW-related and thermal contributions.

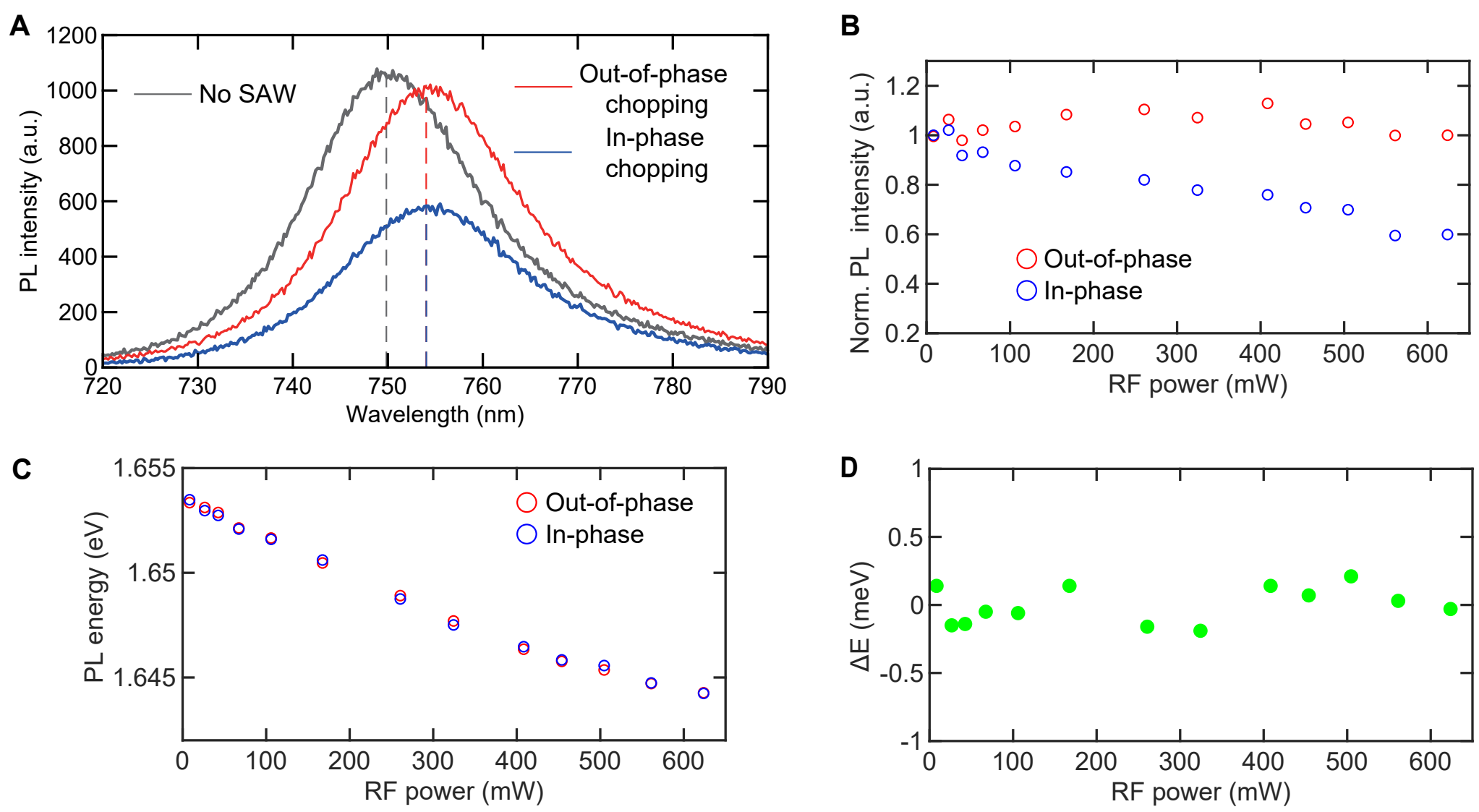

Figure S2: **Modulation-based control measurement for RF-induced heating.** (A) Representative PL spectra measured without SAW excitation and under out-of-phase and in-phase chopping conditions. The optical excitation power is 1 µW, and the representative spectra were measured at an RF power of ~28 dBm. (B and C) RF-power dependence of the normalized PL intensity and PL peak energy, respectively. (D) Difference in PL peak energy between the in-phase and out-of-phase chopping conditions. The small residual energy difference indicates that RF-induced heating provides a dominant contribution to the time-averaged spectral red shift.

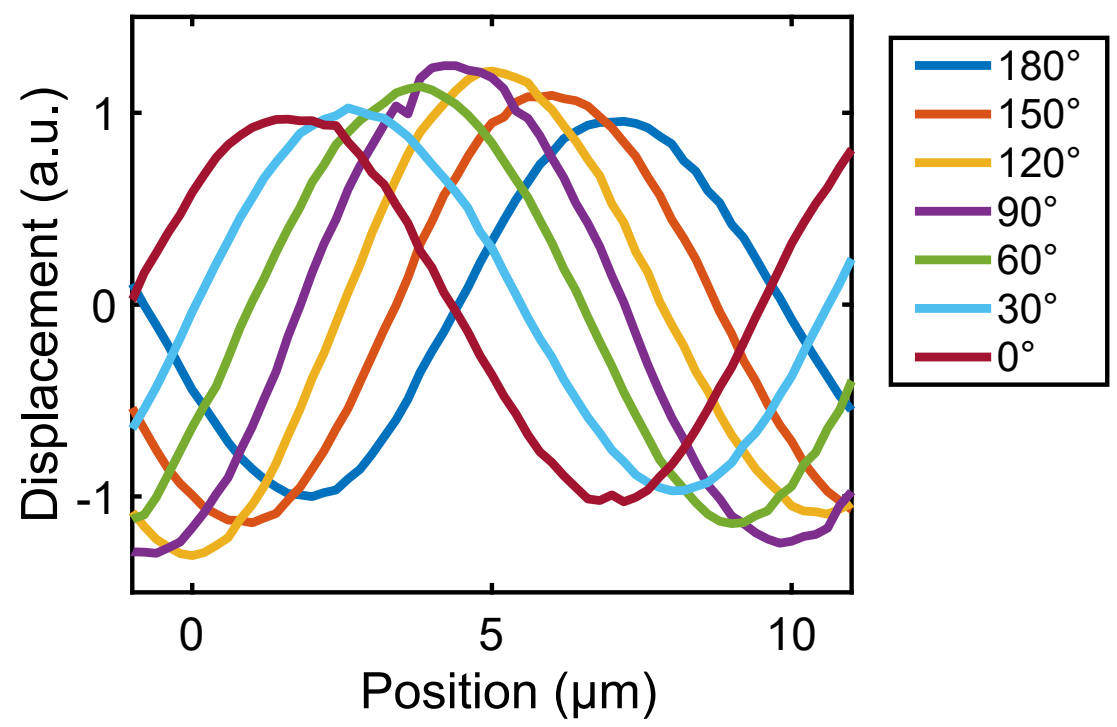

Figure S3: **Spatial profile of the traveling SAW measured using a Michelson interferometer.** The real-space propagation of the SAW was captured over half of an acoustic cycle within an area of approximately 11 μm by performing position-scan measurements of the SAW displacement at each RF phase from 0° to 180°. Spatial profiles of the propagating SAW are obtained, showing a wavelength of approximately 10.6 μm. The propagation direction is from left to right in the figure. We note that, due to the presence of a reflected wave with an amplitude of up to 20% of the incident wave, the SAW amplitude is slightly modulated as a function of RF phase. Such modulation does not occur under ideal single-pass propagation conditions.

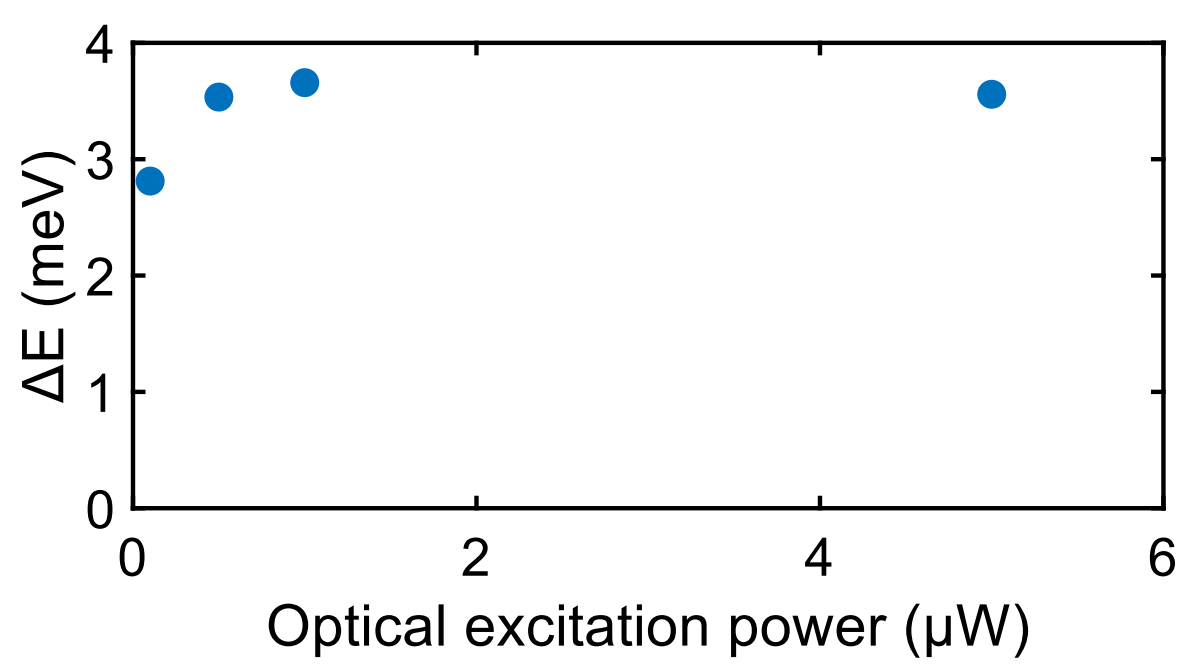

Figure S4: **Dependence of ΔE on the optical excitation power.** The RF excitation power is fixed at 30 dBm. The SAW-induced PL energy modulation is nearly independent of optical excitation power.

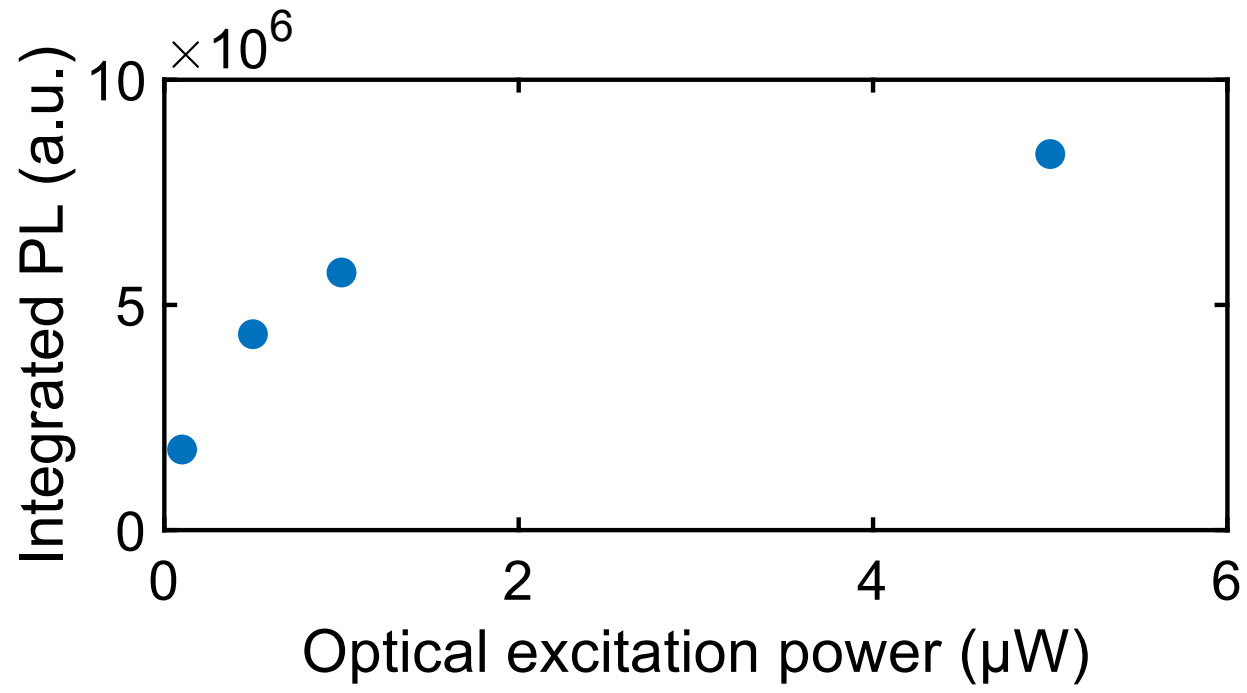

Figure S5: **Dependence of the integrated PL intensity on the optical excitation power.** The RF excitation power is fixed at 30 dBm. The integrated PL intensity saturates at high excitation powers.

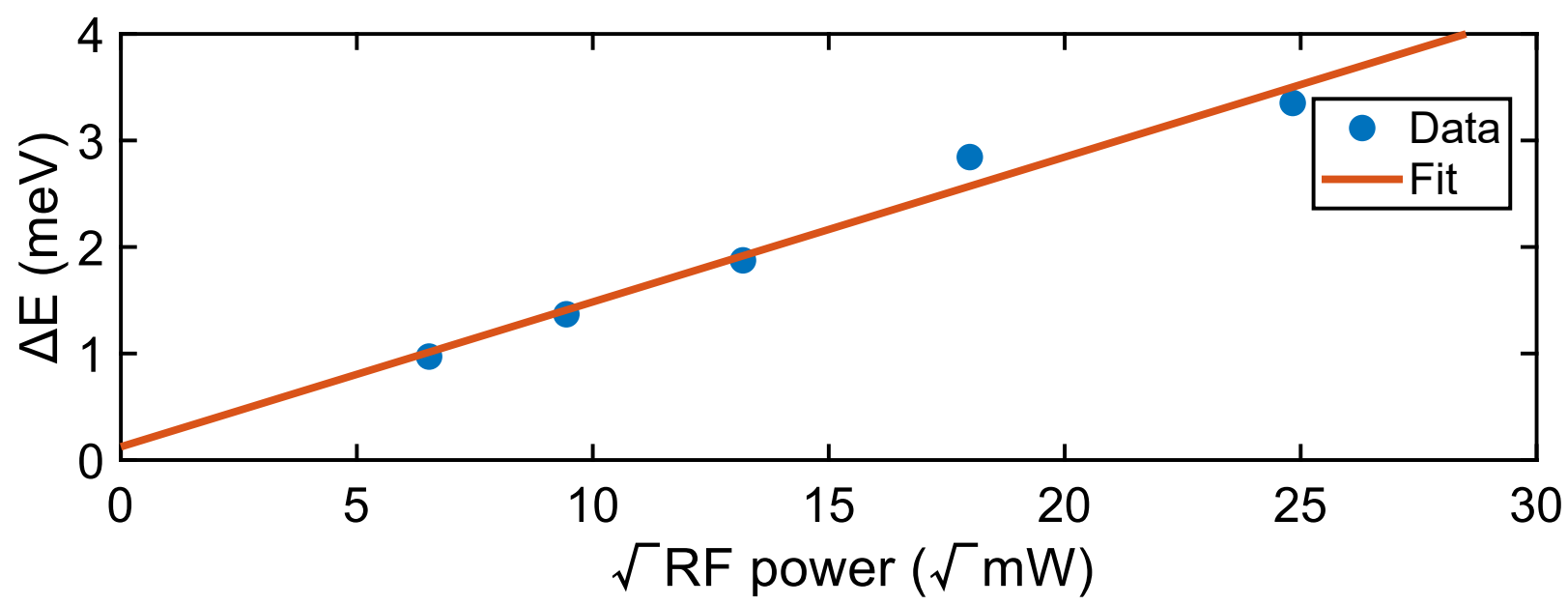

Figure S6: **PL energy modulation ΔE as a function of RF input voltage.** The horizontal axis is the square root of RF power, which is proportional to the RF input voltage. The solid line represents a linear fit.

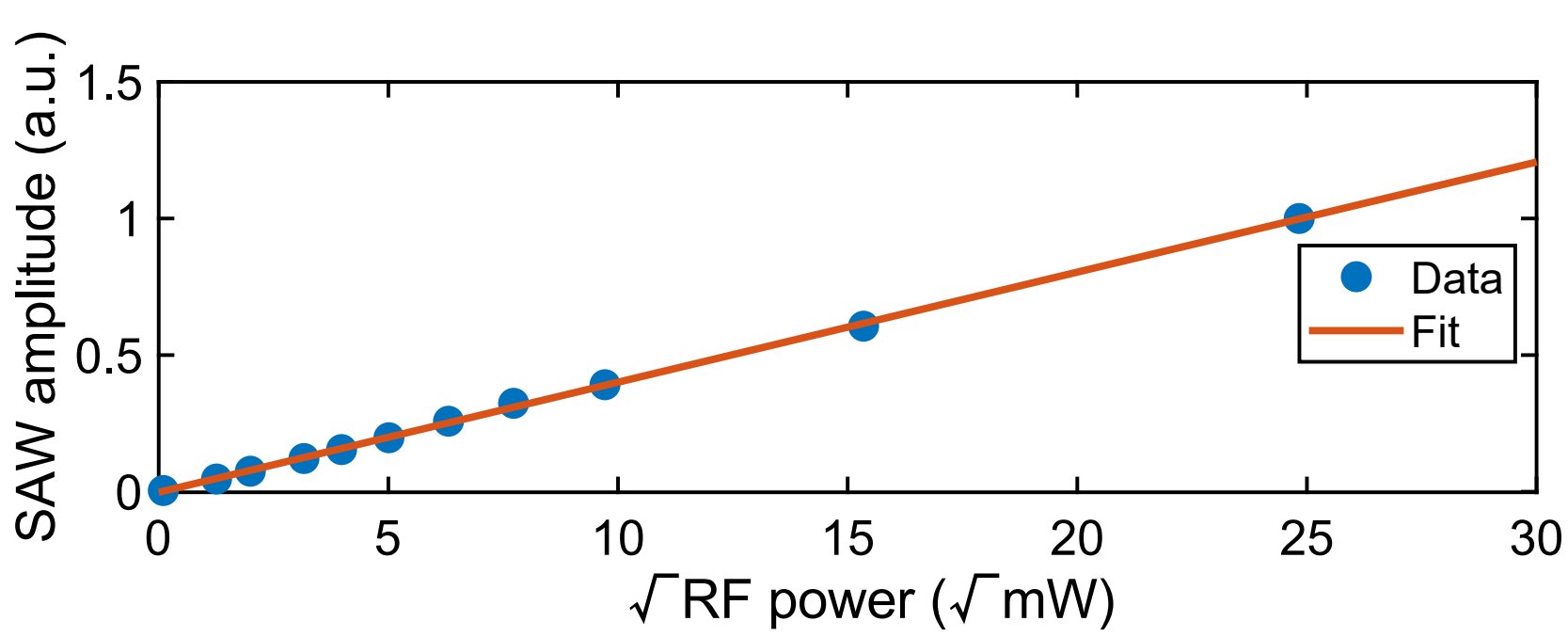

Figure S7: **SAW amplitude as a function of RF input voltage.** The square root of RF power is used as the horizontal axis. The solid line represents a linear fit.

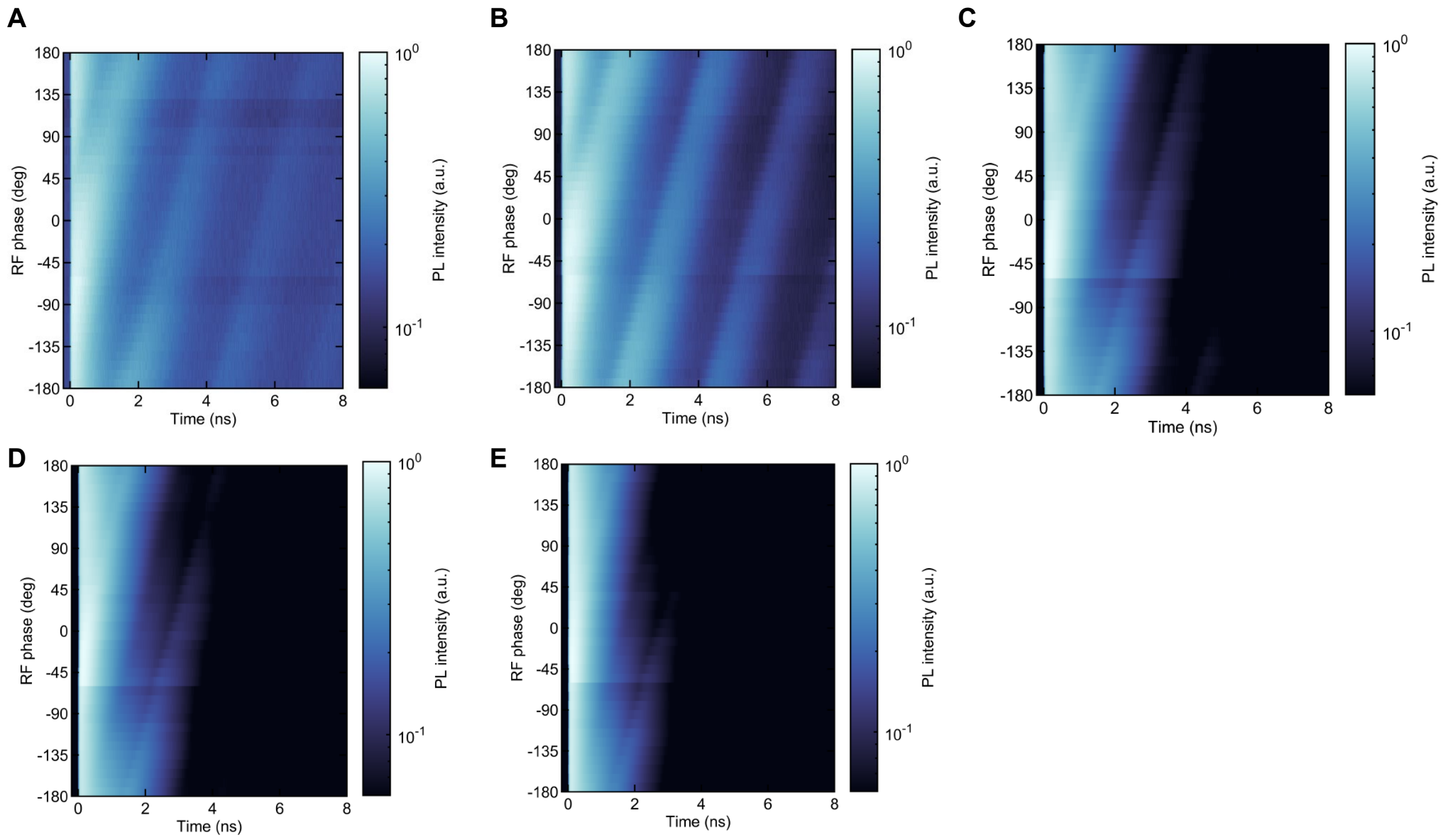

Figure S8: **RF phase dependence of time-resolved exciton PL dynamics.** The RF excitation power is fixed at 30 dBm. The optical excitation powers are (a) 0.05 µW, (b) 0.1 µW, (c) 0.5 µW, (d) 1 µW, and (e) 5 µW.

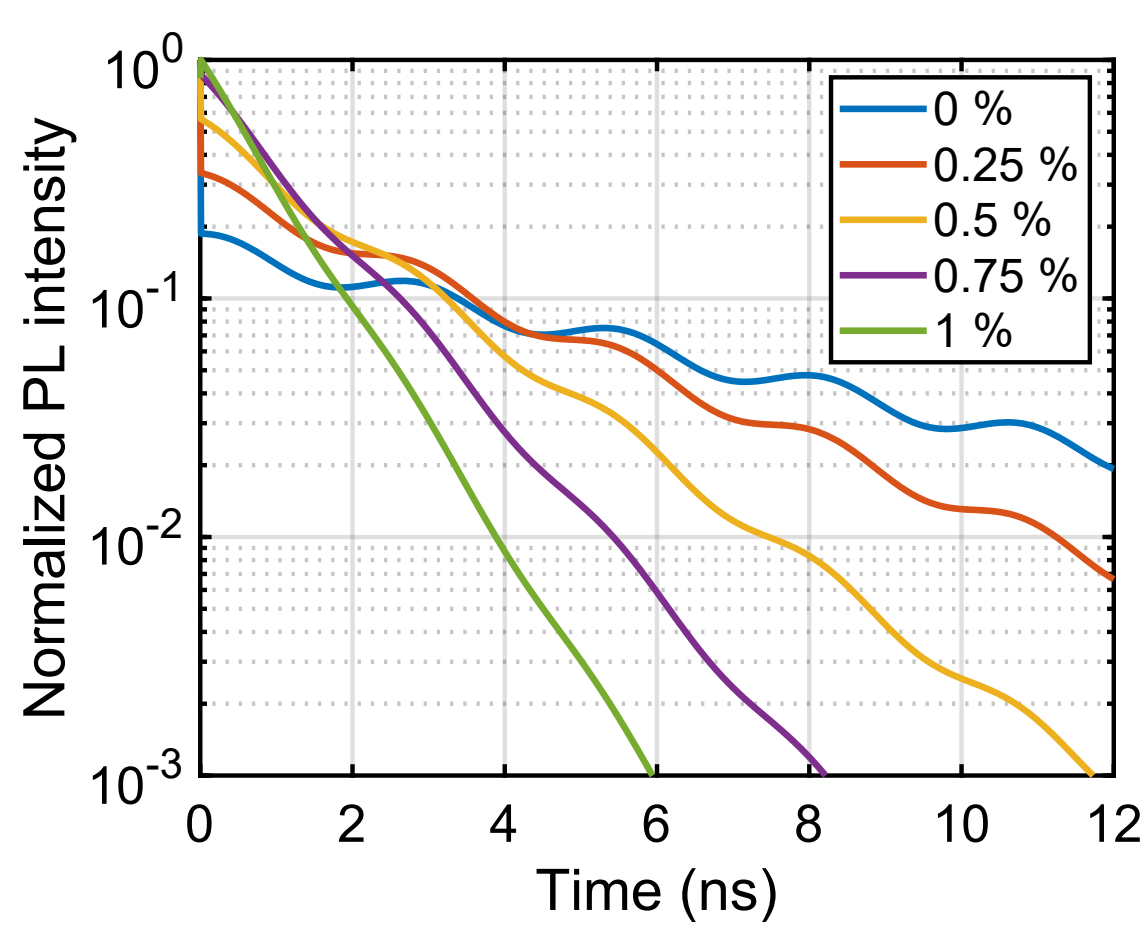

Figure S9: **Simulated PL decay dynamics for different initial tensile strains.** Simulated time evolution of the PL intensity for initial tensile strains of 0%, 0.25%, 0.5%, 0.75%, and 1%. All other parameters are the same as those used in the main text.

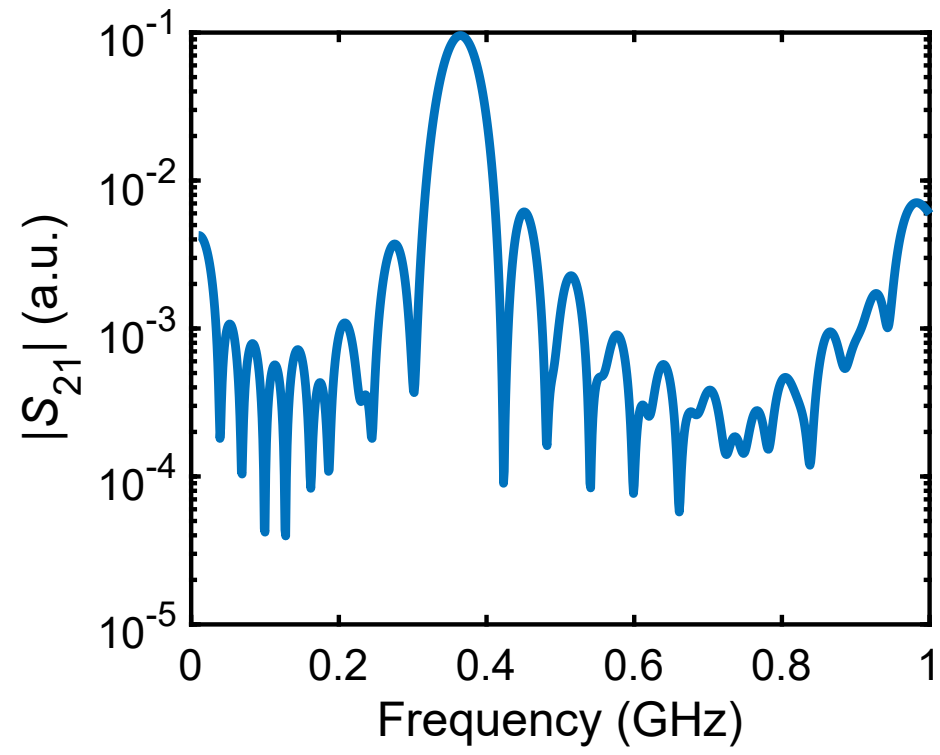

Figure S10: **Transmission spectrum of the SAW device.** The transmission spectrum ($|S_{21}|$) is measured using a vector network analyzer. The resonant frequency is ~365 MHz, and the full width at half maximum (FWHM) linewidth is ~54 MHz.
